# Computational approach for investigating nanoscale interfacial ice adhesion trends

**DOI:** 10.1039/d3ra04854c

**Published:** 2023-12-12

**Authors:** Abhay Vincent, Marie Pervier, Hugo Pervier, Devaiah Nalianda

**Affiliations:** a SATM, Cranfield University College Road Cranfield UK abhay.vincent@cranfield.ac.uk

## Abstract

For developing high performance, low-energy ice protection systems, it is vital to understand the icing physics at the interface of the ice and substrate. Macroscopic experiments have known limitations when it comes to explaining the adhesion characteristics of ice. There is a need to look at the microscale behaviour of ice and how it interacts with the surface it adheres on. The article describes application of molecular dynamics to the ice-substrate problem by modelling two major modes of ice adhesion test – tensile and shear tests, which are used for ice adhesion strength determination. The coarse-grained model of water is nucleated to form ice at the temperature which is designated for ice adhesion test on a macroscopic level. Steered molecular dynamics (SMD) is then applied to the nucleated ice cube to then obtain tensile and shear adhesion strengths over various FCC surface morphologies that represent the crystal structure of metallic substrates. The results obtained from the adhesion simulations are then used to compare the nanoscale trends on ice adhesion to the macroscale ice adhesion trends. The simulation results show that while contact area and temperature variations have similar trends to the observed macroscopic trends, other variations like tensile and shear loading rate variation at the nanoscale are not directly understood from macroscopic interpretations of ice adhesion.

## Abstract 2

For developing high performance, low-energy ice protection systems, it is vital to understand the icing physics at the interface of the ice and substrate. This can be achieved by modelling the interface at nanoscale and then studying the adhesion trends.

## Graphical abstract

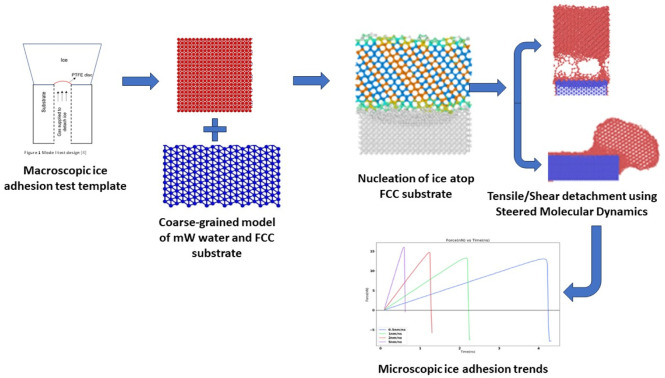

## Introduction

The dangers of icing are well known within the engineering domain. It can lead to hazardous conditions or in extreme cases, serious accidents that can even result in loss of life.^1–4^ Ice protection systems have thus been developed in order to counter the icing that occurs on engineering structures or systems in various civil and mechanical industries.^5–7^ The focus on improving these ice protection systems requires a deep understanding of how ice interacts with substrates. This is achieved by testing the ice adhesion strength of various surfaces that form part of the systems or structures which are exposed to icing conditions. Ice adhesion strength is defined as the maximum force required to detach ice from a substrate surface divided by the apparent contact area.^8^ Traditional methods of measuring ice adhesion strength include the various tensile and shear test methods that have been developed over the years.^9^ The brittle and complex mechanical behaviour of ice under different kinds of loads makes it difficult to understand the adhesion phenomena and predict accurately how ice might behave when subjected to an external force. Study of interfacial ice adhesion has been conducted extensively to understand the nature of ice and it's behaviour with respect to different substrates.^10–13^ It is important to analyse the atomistic ice adhesion behaviour to build a comprehensive model to determine behaviour of ice on various substrates. Nanoscale mechanisms of ice adhesion describe the effect of non-bonded interactions like van der Waals and electrostatic interactions being important to adhesion of ice to a substrate.^14^ An in-depth look at nanoscale fracture mechanics is necessary for our complete understanding of the ice adhesion phenomenon. Macroscale experiments are not the best possible tool when it comes to understanding ice-substrate interactions or adhesion mechanisms precisely because they don't provide access to nanoscale fracture mechanics. The spatial and temporal resolution of macroscale experiments is limited.

The best suited tool for analysing nanoscale interactions of ice and substrate is atomistic modelling and molecular dynamics (MD) simulations. Atomistic water models can capture in more detail the solid–water interfacial region.^15^ It has been used in multiple studies for gaining further understanding of nanoscale ice adhesion mechanisms and interactions with various types of surfaces.^16–19^ Macroscopic ice adhesion trends have been studied in the past.^20–23^ These trends have been mostly consistent across the various studies for different factors that affect ice adhesion like temperature, contact area, loading rates *etc.* The previous literature reviewed for this research work shows that, these ice adhesion testing trends have not yet been studied at nanoscale. This paper describes the development of an ice-substrate atomistic model, based on known tensile and shear ice adhesion tests, using which ice adhesion strength values have been obtained for idealized FCC substrates. The variation in these adhesion strength values are studied with respect to the change in loading rate, temperature, contact area and surface morphology. These trends are then compared to the macroscopic test trends from literature to obtain the similarities or differences between the two scales which is the key contribution to knowledge from this paper. The results from this study can be fed into higher order models like crystal plasticity FEM models for ice adhesion or mesoscale ice formation models. These higher order models can be important tools for optimization of material design for ice protection in the future. The simulations are carried out in the software package called LAMMPS (Large-scale Atomic/Molecular Massively Parallel Simulator).^24^ Visualization of the models and the simulation results is done in the software package OVITO (Open Visualization Tool).^25^ For studying the various ice adhesion trends, a coarse-grained modelling strategy is adopted. The substrates for the coarse-grain model are generic Face Centred Cubic (FCC) surfaces of 4 different morphologies.

## Background

The molecular dynamics simulations conducted in this research work reproduces a tensile and a shear adhesion test and replicate the mode of force application for both these types of tests. Although, many forms of such tests exist in industrial and research practice today, the two tests that this study focuses on, for a baseline to be used in the MD simulations, are the mode I test^26^ and Scrape adhesion test,^27^ both developed at Cranfield University. They have been described below.

### Mode I test

**Fig. 1:**
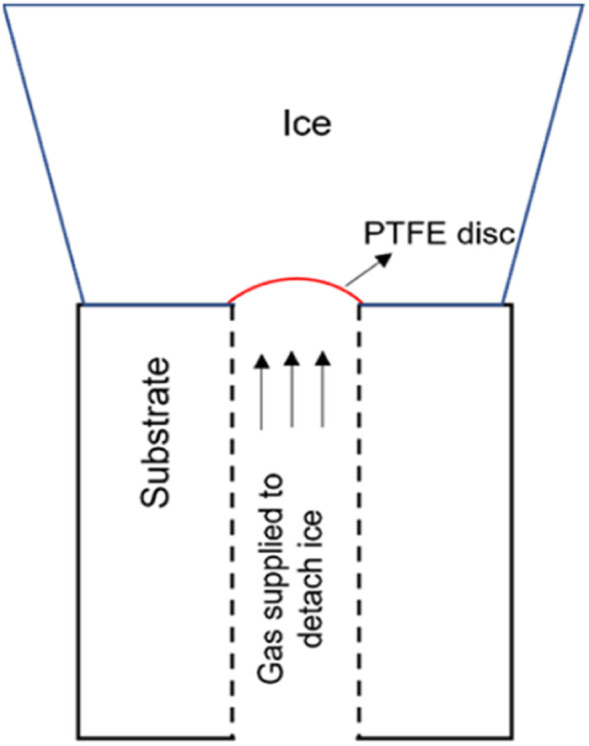
Mode I test design.^8^

This test procedure for ice adhesion has been developed by Hammond.^28^ It is based off the Andrews & Lockington Blister Test. This test process makes use of a hollow cylinder which is covered partially by a small plastic disc. Ice is accreted on the cylinder to a significant enough thickness for the test to be in plane-strain conditions. The pressure is applied to the ice for delamination through the hole in the cylinder. The pressure is increased until the ice breaks of at a value called critical pressure, *P*_c_. The failure can be categorized in 3 different ways: fully adhesive, fully cohesive, or partly adhesive and partly cohesive. Fig. 1 shows the setup for a mode I test with the accreted ice on top of the test substrate with the interfacial plastic disc acting as the defect. Cranfield modifications developed by Hammond includes connecting a vacuum pump in order to keep the plastic disc from falling when the icing tunnel is in operation. The surface of the cylinders faces the oncoming flow and is oriented as such. The plastic disc initiates the crack which means that the dimension of the flaw is known. The load rates can be varied based on the pressure rate applied. Finally, the calculation of the fracture energy needed to break the ice of the substrate can be achieved by the means of an analytical solution and using this method, the fracture toughness, and the tensile strength of ice can be ascertained as well. More details on the test procedure and setup, calculation of fracture energy and adhesion strength can be found in (ref. 26).

### Scrape adhesion test

**Fig. 2:**
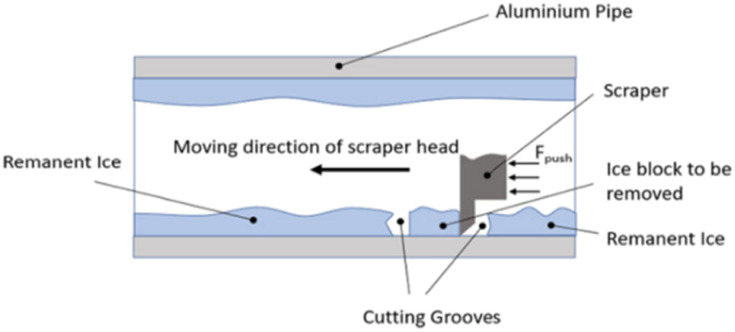
Scraper test design.^27^

The setup for the scrape adhesion test consists of a pipe, on which ice is accreted. A heated cutter is used to make a slot in the accreted ice such that a keyed guide shaft arm consisting of a scraping blade can be used for delamination of the ice. A load cell is used for capturing the recorded force values and a pneumatic actuator provides the requisite force to detach ice from the pipe surface in a shear manner. Fig. 2 shows the detailed design of the scrape adhesion test for a cylindrical pipe sample. Further details for the scrape test setup and test procedure can be found in (ref. 27).

## Methods

### Atomistic modelling

The crucial step in atomistic modelling of ice adhesion is to determine the correct water or ice model for simulation. Since, the intent of this research was to study the adhesion trends as they occur in a test setup, it is important that the ice is nucleated on the surface rather than beginning with a pre-defined ice block of any set description. Keeping in mind the simulation timescales required for water nucleation to ice using complex, higher order water models like TIP4P^29^ and SPC,^30^ the strategy to use coarse-grained modelling for this work was made. The coarse-grained models are created to simulate the behaviour of complex systems using a simplified or coarse-grained representation of the system. By decreasing molecular details in the modelling, these models can study system behaviours for much longer simulation times. Monoatomic water (mW) potential^31^ is used for modelling the water molecule in this work. In the coarse-grained mW model, each H_2_O molecule is treated as a single particle interacting through anisotropic short-ranged potentials (the Stillinger–Weber potential). Although explicit hydrogen atoms and electrostatic terms are not included, the mW model can correctly describe the thermodynamic properties and phase behaviour of water in bulk and on substrate. mW water model is able to replicate the melting point of hexagonal ice to a closer degree (274.6 K) when compared to other available atomistic models of water. It has been reported to reproduce the structural, thermo-dynamical properties of ice and water, and has been extensively applied to study the crystallisation of bulk water and nanodroplets as well as on different substrates.^32^

**Fig. 3:**
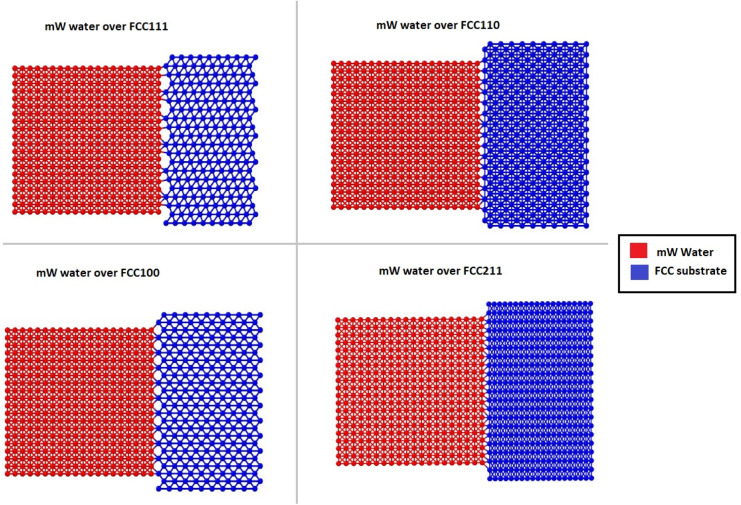
Four ice-substrate atomistic models to represent the different packing of atoms in the FCC substrates pre-equilibration.

The different atomistic models for the substrate in the coarse-grained model are defined as four different crystallographic planes of a generic FCC crystal: {111}, {100}, {110}, and {211} surfaces. These models exhibit significant differences in terms of atomic roughness and the symmetry of the outer crystalline layer. The surfaces were created using the Atomic Simulation Environment (ASE) package in Python.^33^ Due to the difference in surface morphology between the four different types of FCC crystallographic surfaces, the sizes of the models vary slightly along with the number of atoms that range from 8200 to 9250 for the four models. Also, important to note that for most studies on ice adhesion test microscopic trends, FCC111 was chosen as the main representative surface to be used with the water slab. A larger model was also created with the FCC111 lattice orientation, in order to study the effects of a larger contact area on the adhesion strength at the microscopic level. Both tensile and shear simulations were performed on this larger model. The lattice constant was chosen to be around 4.05 for all the surfaces to ensure a good packing density of atoms within the substrate structure and to have a closer match to Aluminium which is the standard material used in Mode I tensile adhesion tests.^26^ For the water structure, a random lattice of atoms is defined in LAMMPS with dimensions of 10 × 10 × 10 A^0^ to give around 4000 atoms in total and surface contact area of 1 nm^2^. This structure is then minimized and equilibrated at 300 K within the canonical ensemble (NVT) for 20 ns to generate the final structure which can be used for the coarse-grained combined model. Fig. 3 shows the four combined water and FCC model used in the coarse-grained models.

The individual FCC lattices have different configurations resulting in different packing densities hence a different number of atoms. The water structure is not directly placed on top of the substrate but separated by a distance of 2 A^0^. The process of energy minimisation is used to bring both the structures in contact.

### Coarse-grained simulations

#### Atomic interactions

Table 1 describes the various potentials used for the coarse-grained simulations.

**Table 1:** Potentials for various interactions

Interactions		
Surface–surface	Water–water or Ice–ice	Surface–water/ice
None (inert surface)	mW potential^31^	LJ potential^34^

The coarse-grained potentials have no surface–surface interactions since they are supposed to be generic FCC structures to allow the water model to nucleate effectively on the surface. The surface–water/ice interactions are primarily defined by aluminium–oxygen interactions.^35^ The aluminium–hydrogen interactions are infinitesimal in comparison and thus are ignored for these simulations. The Lennard–Jones (LJ) potential for these interactions is based on the values defined in Mao *et al.*^34^ The main two parameters for the LJ interaction are given in Table 2.

**Table 2:** LJ Potential for Al–O interactions

Parameter	Value
*ε*_Al–O_	0.053 eV
*σ*_Al–O_	2.858 Å

#### Simulation settings

The coarse-grained model made use of the ‘real’ units. Since the mW potential allows for larger timesteps within the simulations, a value of 10.0 picosecond (ps) for the equilibration and nucleation simulations and 5.0 ps for the force-probe simulations was used. The standard temperature selected for the ice-substrate simulations was 251 K (−22 °C). The models make use of the steepest descent algorithm for minimisation in order to relax the atomistic structures. The same equilibration process is carried out throughout for uniformity. The structures are initialised with an assigned temperature of 273 K (0 °C) and then equilibrated using the NVT ensemble for 50 ns. The NVT ensemble was selected for both the models as the appropriate ensemble to perform force-probe MD simulations,^16^ with use of the Nosé–Hoover coupling method to maintain the simulation temperature.^36,37^ The effect of the selected thermostat coupled with the NVT ensemble needs to be acknowledged before proceeding to the finer details of the modelling within this study. The use of the NVT ensemble precipitates that the results obtained from this work should be treated as figures of merit with which to investigate local effects. It is important to note that, the results do not show a one-to-one correspondence between adhesive strength obtained from the ice-substrate simulations in this work and those measured by an experiment at a much lower loading rate. The NVT ensemble with the coupling effect of a strong heat bath ensures that the model does not melt away or the simulation box doesn't vaporise under extremely high loading rates.

#### Process for coarse-grained simulations

In order to perform the ice adhesion test, the starting point was the mW water model which is equilibrated in a separate simulation and the final equilibrated structure is combined with the substrate to form the final model. After this step however, a nucleation simulation is required which involved a shock-quenching with an immediate drop of temperature from 273 K to 180 K at the very start of the simulation resulting in nucleation and subsequently a rise in temperature to 251 K. This technique ensures that the water structure nucleates into ice and subsequently while heating up to 251 K which is the target temperature for the simulations, the ice doesn't melt into water.

#### Nucleation process

In order to nucleate the water into ice, the simulations follow the strategy adopted by Fitzner *et al.*^38^ The water structure is first equilibrated at 273 K. After the structure is stable, the structure is then quenched from 273 K to 180 K which represents a drop in temperature of 93 K. This process is done at a cooling ramp of 0.5K ns^−1^ and the total time for this process is around 194 ns which includes some additional 8 ns for stabilization post-quenching. There are minor changes for the different lattice models with the major change being the additional time added post quenching for stabilization which varies from 8 ns to 20 ns. The quenching results is a change of thermodynamic phase from water to ice and a re-ordering of the atomic structure. A sudden and sharp drop in potential energy characterizes this phase change.^38^ The most natural path for the ice crystal to grow preferentially is from a basal plane.^14^ This is possibly the reason why a small layer of water is observed in the coarse-grained models post the nucleation process. The prismatic plane is not considered a preferential growth plane for ice on the substrate. For the ice crystal to grow into a prismatic alignment during the simulation is significant but also slightly suspect as it deviates from the natural tendency of crystal growth for ice through the basal plane. The reason for this deviation could be:

Size effect: the cell is so small the amount of water molecules introduced can only grow in that direction for the box dimensions chosen.

• Geometric size effect: similar to the size effect but pertains more to the geometry of the simulation box which is in effect a square.

• Limitations of coarse graining: the unusual ice crystal growth could be a consequence of interactions at the interface being not captured well enough or being too non-directional, which leads to prismatic growth.

**Fig. 4:**
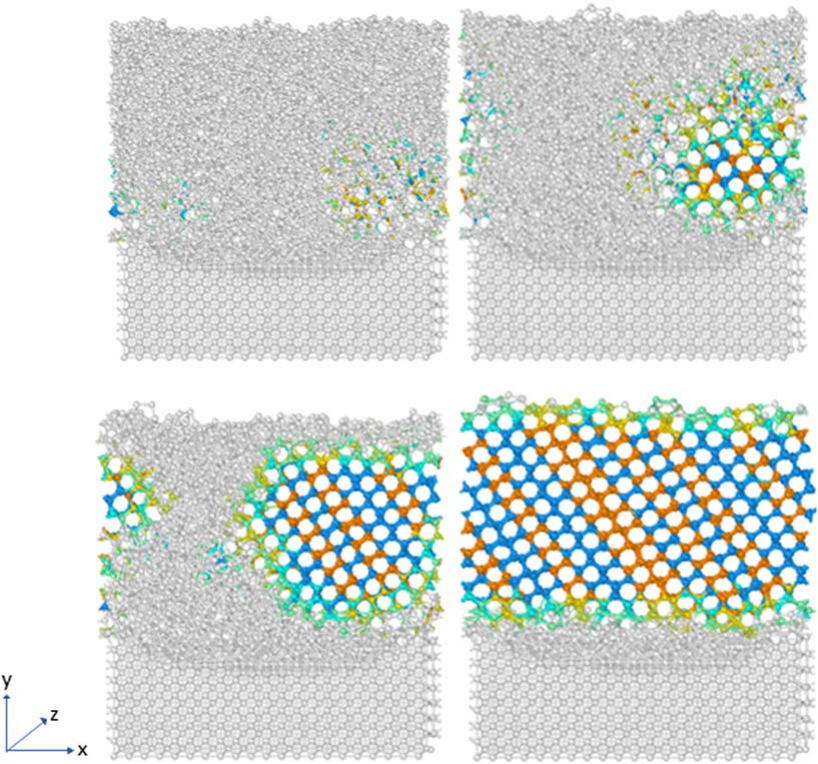
Nucleation process for FCC100-mW water model.

This particular approach with the coarse-grained models is significant in capturing this effect of unusual ice crystal growth on some of the substrates. This also reinforces the perceived complexity in modelling ice-substrate adhesion problem. In order to have a more comprehensive and detailed look at the ice adhesion to various substrates problem and extract useful data from it, there needs to be a consideration of other types of contact between ice and the substrate like basal and pyramidal contact with each relevant substrate surface. Fig. 4 shows the process that the water molecules reorganise themselves into the hexagonal close-packed structure associated with ice during the nucleation process.

#### Force application

**Fig. 5:**
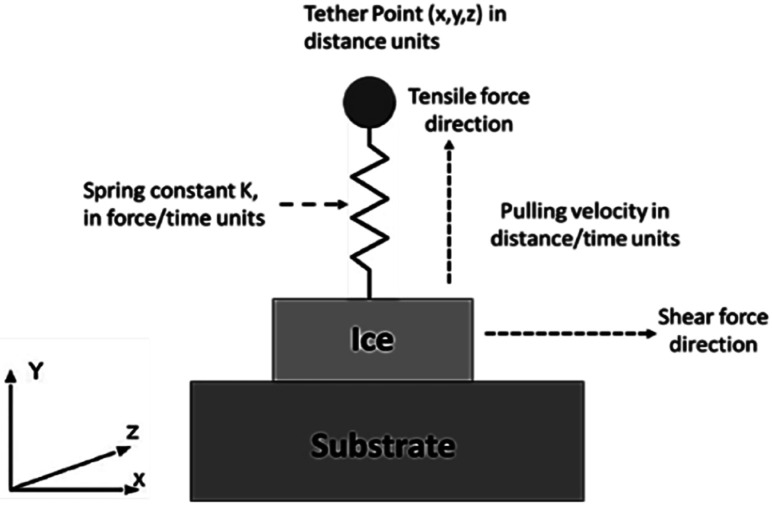
Steered molecular dynamics applied to ice-substrate problem.

**Fig. 6:**
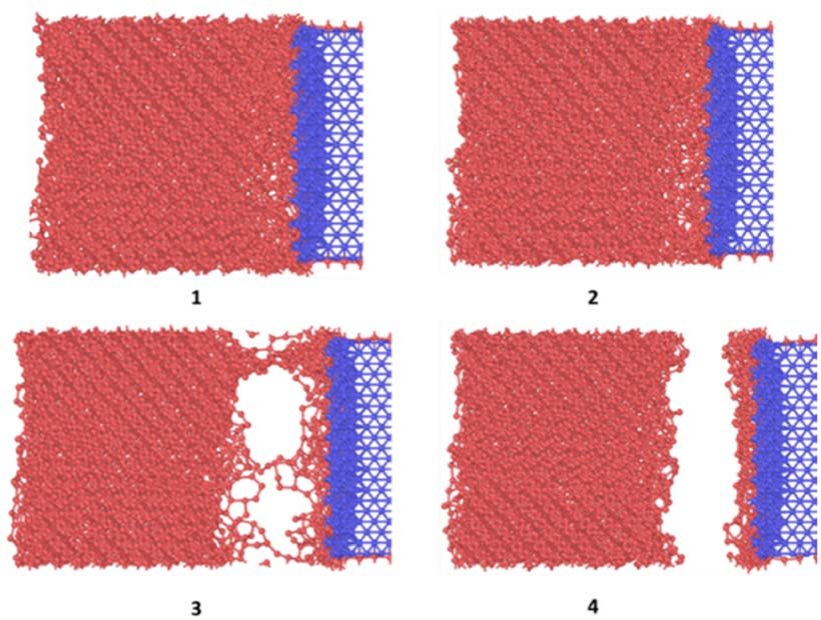
Tensile detachment of ice in cohesive manner from FCC100 substrate.

The most suitable option within LAMMPS for application of tensile and shear force was determined to be steered molecular dynamics using ‘fix smd’.^39–41^ Using fix smd, the centre-of-mass (COM) of the ice model is attached to a virtual spring within the simulation which is tethered to a point in the direction of the push/pull. The spring is assigned a chosen value of spring force (force/distance units) and a velocity (distance/time units). The ice model is then pulled away from the substrate and the resultant force is then captured which is determined to be the requisite force for ice delamination as shown in Fig. 5. In order to explore the effects of various loading rates, the spring force were varied and so was the assigned velocity. The selection of the applied tensile or shear force values through Steered Molecular Dynamics (SMD) is based on the lowest possible loading rate for which the ice delamination can be observed from the substrate when balanced within reasonable computational time. For the standard simulation, the spring force value was 10 kcal mol^−1^ Ang^−2^, and the assigned velocity was 0.5 nm ns^−1^ for the tensile delamination. For the shear delamination, the applied spring force was 10 kcal mol^−1^ Ang^−2^ and 15 Kcal mol^−1^ Ang^−2^ with a speed of 10 nm ns^−1^ and 15 nm ns^−1^ at both spring constants. Lower values failed to shear the ice from the substrate completely. It is a trial-and-error process to find the correct force for delamination with a reasonable computational time and processing effort. For lower loading rates, delamination of ice maybe observed but the excessively long computational time required makes it undesirable while working on the parallel processing queues. In order to perform fix smd, it is essential to have a counter force acting on the substrate to prevent it from being pulled along with the ice model. This was provided by the use of ‘fix spring/self’ which constrains the substrate model in place. The simulation box is expanded in the direction of the pulling force in order to observe the effect of the ice detachment. Fig. 6 shows the process of the detachment of the ice nucleated on top of the substrate on application of the force through fix smd. The streaming velocity of ice due to application of a tensile or shear force was accounted for by using the compute temp/profile command in LAMMPS and then modifying the NVT fix accordingly. The removal of the spatially-averaged velocity field by this method is essentially computing the temperature after a “bias” has been removed from the velocity of the atoms.

## Results and discussions

### Atomistic ice detaching and shearing mechanics

Under the applied load, the ice substrate is delaminated from the substrate. This force is captured by the 7-vector output produced by the ‘fix smd’ within LAMMPS which gives the following quantities: the *x*, *y*, and *z* component of the pulling force, the total force in direction of the pull, the equilibrium distance of the spring, the distance between the two reference points, and finally the accumulated Potential mean force, PMF (the sum of pulling forces times displacement). The trends for adhesion tests were studied using idealized substrates and coarse-grained models. This facilitated faster computational times allowing for more flexibility and evaluation due to the rapid dynamics of potentials like mW. Although mode I test were used as a rough template for the simulations, it is clear that the models require further development for them to be an accurate replication. This is especially true in terms of the force replication. Mode I test employs pressurized Nitrogen through a hole in the substrate to dislodge the ice accreted on it.^8^ This was particularly challenging to replicate and needs further investigation in terms of the MD methods currently available. The trends observed have been discussed in more detail below.

### Ice adhesion strength value

The ice adhesion values found by this research work significantly higher than the ones observed at the macroscopic level. This is consistent with previous work done on ice adhesion in MD simulations.^16,17,42^ The values are way higher than those observed traditionally in macroscopic experiments which are around 1 MPa.^8,43–46^ This is mainly due to the loading rates used in molecular dynamics which are of 6–7 orders of magnitude higher than their macroscopic counterparts.

### Variation of adhesion strength with tensile loading rate

**Fig. 7:**
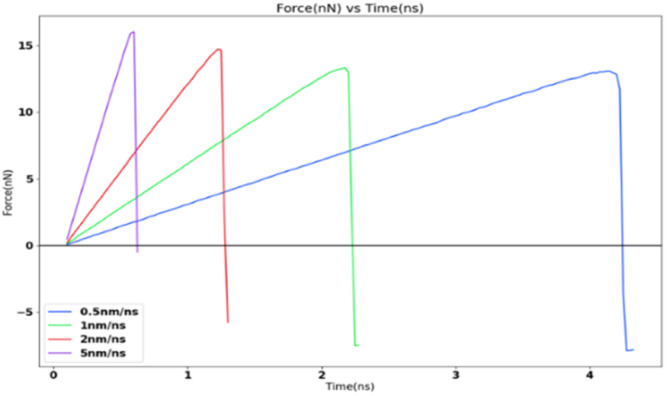
Tensile force variation at four different loading rates.

In order to study the effects of the tensile loading rate of the adhesion strength, the assigned spring velocity which represents the loading rate (displacement/time) was varied on the FCC111-ice combined coarse-grained model with lesser apparent contact area of 1 nm^2^. The temperature of the simulation was maintained at 251 K for all simulations and the NVT ensemble was used for the force-probe simulations. Fig. 7 shows the variation in the tensile force at different loading rates from 0.5 nm ns^−1^, 1.0 nm ns^−1^, 2.0 nm ns^−1^ to 5.0 nm ns^−1^. The value of adhesion strength and shear force obtained for these force-probe simulations are reported in Table 3. The values show an increase with increasing loading rates.

**Table 3:** Tensile force and adhesion strength for different loading rates

Spring velocity (nm ns^−1^)	Peak force (nN)	Adhesion strength (MPa)
0.5	13.07	13 071.77
1.0	13.32	13 322.37
2.0	14.72	14 726.48
5.0	16.02	16 022.20

**Fig. 8:**
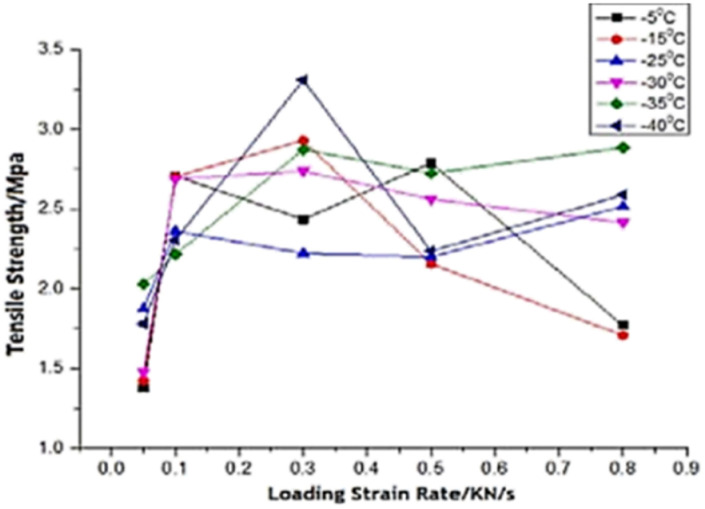
Relationship between tensile strength and loading rate.^23^

The simulation results show that the ice adhesion strength at nanoscale increases with increase in loading rate. Gold^20^ clearly states that the strength of ice depends on the rate of loading and the manner of the load application to the substrate apart from other factors involved in the process of failure. As noted by Kasaai *et al.*^47^ ice exhibits elastic behaviour at low strain rates and brittle behaviour at high strain rates. This in turn means that adhesive failure is more likely at low strain rates and cohesive failure at higher strain rates. This is observed in the results from this research work as well with all the coarse-grained models yielding cohesive or mixed failures. The trend of ice adhesion strength with tensile loading rate is not clear from macroscopic experiments as noted by Meng *et al.*^23^ The tensile strength shows little increment with the increase in loading rate. The tensile strength of ice reaches a maximum value at a specified loading rate. The tensile strength after this point, decreases with the loading rate continuing to go up. The tensile strength of ice reaches the maximum 3.3070 MPa at −40 degrees Celsius and the loading strain rate 0.3 kN s^−1^ (Fig. 8). Thus, it would be objectively wrong to state that the trend observed by the molecular dynamics simulations is actually the case in reality.

### Variation of adhesion strength with shear loading rate

The ice structure was detached under the applied loading although the fracture is observed to be cohesive with a considerable residual layer left on the substrate. The strong interaction between the substrate and the ice atoms which is modelled after the aluminium–oxygen interactions using the LJ potential leads to a completely cohesive break.

**Fig. 9:**
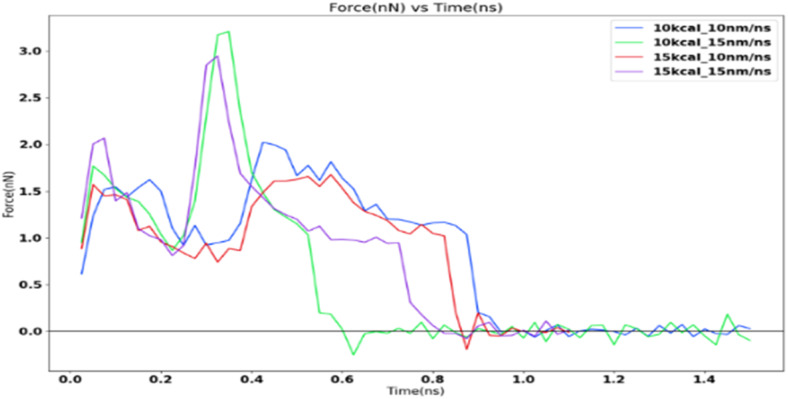
Shear adhesion force at various loading rates.

The force profile as shown in Fig. 9 is reminiscent of the saw-tooth pattern which is observed in shear test force profiles as observed by Xiao.^16^ The highest peak force was obtained for the 10 kcal spring constant and 15 nm ns^−1^ simulation. This is around 3.2 nanonewtons (nN). The corresponding shear adhesion strength was 3207.53 MPa. This is considerably higher than the shear strengths observed in actual macroscopic experiments around 500–2500 kPa.^8^ This is to be expected due to the higher loading rates in molecular dynamic simulations which is around 6–7 order of magnitudes than the macroscopic experiments.

**Fig. 10:**
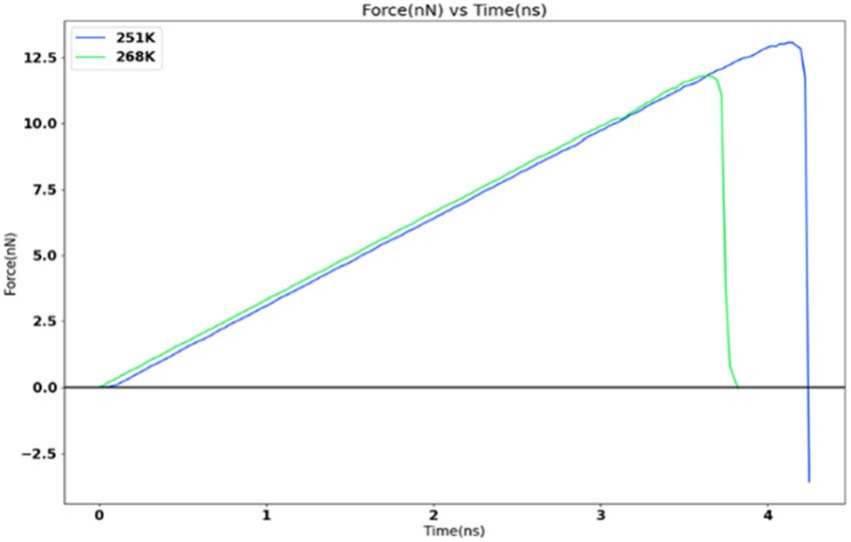
Sequence of ice delamination under shear load.

Fig. 10 shows the sequence of ice detachment from the substrate under the applied load. The trend from the simulation shows that the increase in shear loading rate increases the adhesion strength. However, previous studies on analysis of ice shear strength with varying shear rates in experiments have stated that the peak shear strength slightly decreases with increasing shear rates.^48^ Solid ice is a kind of typical elastoplastic material. When the shear rate is slow, the ice crystal has enough time to shear slip, and it presents ductile failure characteristics. More loading rate data points are required to obtain a holistic picture of shear strength variation for ice with loading rate at the microscale.

### Variation of adhesion strength with temperature

**Fig. 11:**
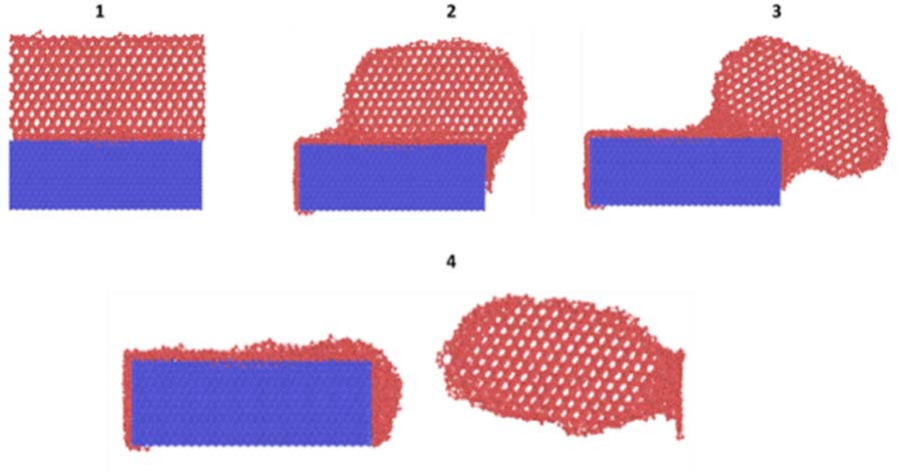
Tensile force variation at two sub-zero temperatures.

In order to study the variation of adhesion force and strength with temperature at nanoscale, the FCC111-ice combined model with lesser apparent contact area (1 nm^2^) was subjected to force-probe simulations with the same force and loading rate of 10 kcal mol^−1^ Ang^−2^ and 0.5 nm ns^−1^ at two different temperatures of 251 K and 268 K. These temperatures are similar to two different temperature settings that can be used in the mode I test at Cranfield Icing tunnel. The result from this simulation is reported in Fig. 11. There is a significant decrease in the adhesion force and strength when the temperature is increased from 251 K to 268 K. This is consistent with the observations from the Mode I test results.^8^ The adhesion strength value changes from 13 071.77 MPa for 251 K to 11 806.89 MPa for 268 K force-probe simulations.

The simulation results show an increase in adhesion strength as the temperature is reduced. The literature study has shown contradicting results regarding this trend. As summarized in (ref. 47), various studies have shown different relationships between ice adhesion and temperature. Studies like Druez *et al.*^49^ reported a linear increase of adhesion strength of ice with reduction in temperature. This is consistent with the results observed for the various temperature settings used for Mode I test in the Cranfield Icing Tunnel.^8^ It was noted by Lack *et al.*^50^ that lower freezing temperature resulted in lower ice adhesion strength which is in direct contradiction to the observed trend in this work. Landy and Freiberger^22^ indicated that the variation in temperature may have two distinct effects based on the thermal coefficients of the ice and substrate:

• Ice adhesion strength should decrease with an increase in temperature if the thermal expansion coefficients of ice and substrate are considerably different from each other.

• if the difference in thermal coefficients of ice and substrate is not appreciable, the ice adhesion strength will vary inversely with the absolute value of the difference between the thermal expansion coefficients. The latter case practically never happens as per the study conducted.

### Variation of adhesion strength with contact area

**Fig. 12:**
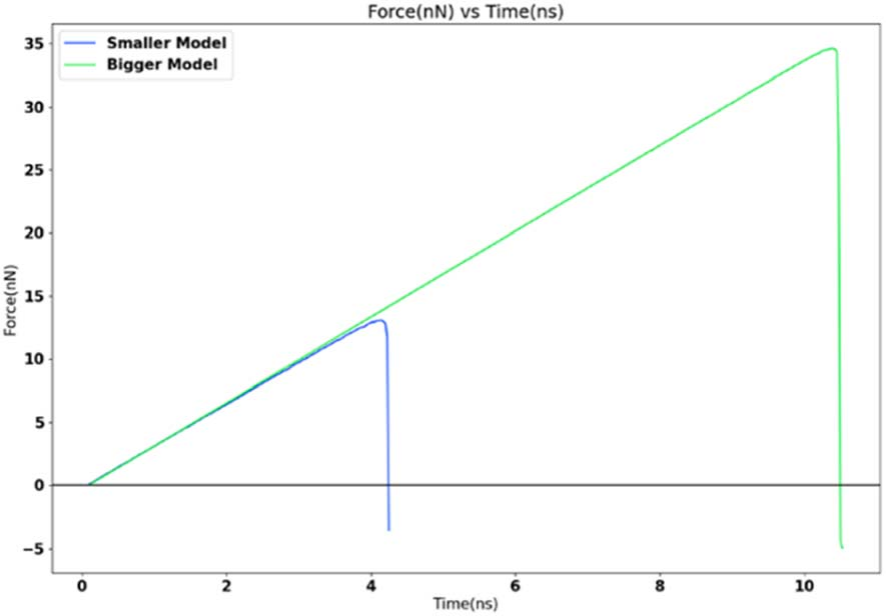
Tensile force variation with apparent area of contact.

For studying the effect of contact area variation, two FCC111-ice coarse-grained models with varying dimensions and contact area (1 nm^2^ and 16 nm^2^) were subjected to a tensile load at 251 K. The tensile load was applied *via* SMD in LAMMPS with a spring force constant of 10 kcal mol^−1^ Ang^−2^ and 0.5 nm ns^−1^ spring velocity. The increase in apparent area of contact shows a clear decrease in the adhesion strength and significant increase in the detachment force as seen in Fig. 12. The adhesion strength varies from 13 071.77 MPa for 1 nm^2^ model to 8467.56 MPa for 16 nm^2^ model with larger contact area.

The adhesion strength depends on the contact area^21^ and is found to increase with a decrease in interfacial area. This trend is found to be consistent in the nanoscale simulations done as part of this research work.

### Variation of adhesion strength with surface morphology

**Fig. 13:**
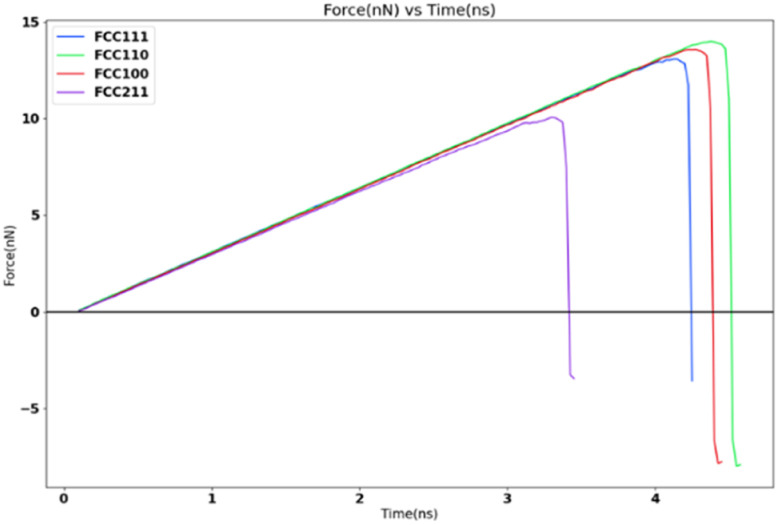
Tensile force variation with four different surface morphologies.

In order to study the effects of surface morphology on the adhesion strength at nanoscale, a tensile load is applied to the four different coarse-grained FCC lattice models with lesser apparent contact area (1 nm^2^) under the same simulation settings and temperature of 251 K. The tensile load is defined by the spring force constant of 10 kcal mol^−1^ Ang^−2^ and 0.5 nm ns^−1^ spring velocity. Fig. 13 shows the variation in the tensile force for the four surfaces, the values are captured from Table 4.

**Table 4:** Tensile adhesion strength and peak force for four different surfaces

Surface	Peak force (nN)	Adhesion strength (MPa)
FCC211	10.06	10 067.2
FCC100	13.57	13 572.2
FCC110	13.98	13 989.9
FCC111	13.07	13 071.7

**Fig. 14:**
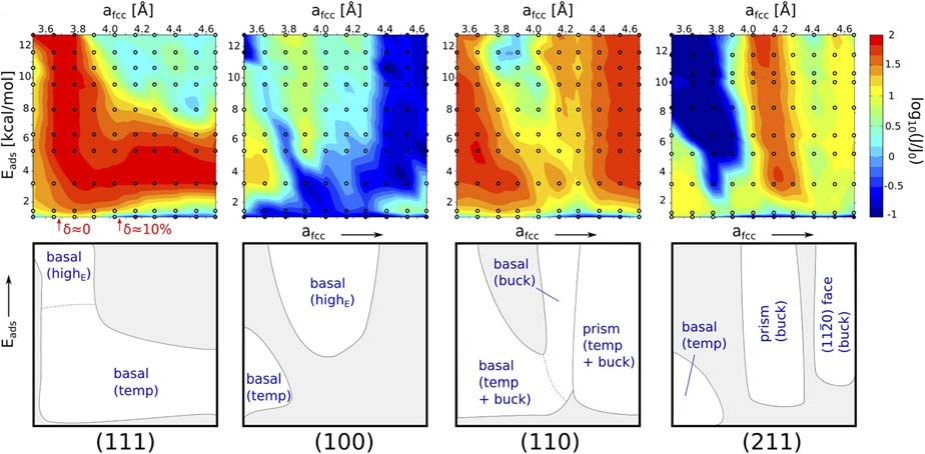
Heat maps representing the values of ice nucleation rates on top of the four different surfaces and sketches of the different regions (white areas) in the space with significant enhancement of the nucleation rate.^37^

The variation of ice adhesion strength with surface morphology is quite a complicated trend to explain. The variation in surfaces allowed for an interesting facet for exploration with regards to study of ice adhesion strength. As noted by Fitzner *et al.*,^38^ the nucleation dynamics of water into ice over the different FCC lattices shows the following trend:

• The different FCC surfaces are shown to mostly promote heterogenous nucleation compared to homogeneous nucleation.

• The {111} and {110} surfaces were seen to promote ice nucleation over a much broader range than the {211} and {100} surfaces.

• It was also noticed that surface symmetry alone was definitely not enough to account for such a difference. In fact, the {111} and {110} surfaces possess different symmetry (hexagonal and rectangular respectively).

• Fig. 14 shows the various regions with respect to the different surfaces that nucleate the ice. This analysis was performed by Fitzner *et al.*^38^

This might explain the variation observed with the adhesion strength with the FCC110 surface showing highest ice adhesion strength with the FCC111 showing the second highest. However, this needs further study. There is no literature found in the macroscopic regime with respect to this trend which is understandable since this work cannot be conducted with traditional ice adhesion experimental procedures.

## Conclusions

The ice adhesion trend analysis was conducted with the use of molecular dynamics simulation. Several variations of these simulations were conducted to study the variation of the ice adhesion strength with loading rates, contact area, temperature, and surface morphology. More data points in general with respect to loading rate in both shear and tensile regimes, contact area and temperature would make for a better and more comprehensive future study. This work has tried to co-relate these trends observed using MD simulations with the trends at macroscopic level. Some of the trends are clearly similar while others need more exploration. It's important to note that research work clearly shows the difficulty in the transability of results from the macroscopic to the nanoscale regime using MD simulations. The applied force for delamination in these simulations operate as a figure-of-merit rather than actual atomic forces. Certain variables like stress and strain do not directly translate at the nanoscale level from the macroscopic level. Usually, it's not virial stress that is computed in these simulations. The strain rates are several degrees higher than the macroscopic regime and thus the results are not one-to-one. However, even with these limitations on the creation of atomic models and the results obtained, it is still worthwhile performing and further exploring the ice-substrate problem using molecular dynamics. The results although not accurate when compared to macroscopic experiments, can still be utilised to create mesoscale models (for *e.g.*: based on crystal plasticity finite element method (FEM) modelling). These models can be used to study in greater depth, the behaviour of ice on various substrates which is not possible from macroscopic experiments or even engineer more specific substrates. Size effects to study how change in simulation box size, aspect ratio and orientations affect the adhesion strength results and nucleation dynamics is an interesting path for further work. Thermal expansion coefficient study and analysis of structural changes of ice with respect to change in temperature would also be of interest for future work in refining this ice-substrate model study.

## Conflicts of interest

There are no conflicts to declare.

## Supplementary Material
